# Hippocampal neurophysiology is modified by a disease-associated C-terminal fragment of tau protein

**DOI:** 10.1016/j.neurobiolaging.2017.07.005

**Published:** 2017-12

**Authors:** Francesco Tamagnini, Darren A. Walsh, Jon T. Brown, Marie K. Bondulich, Diane P. Hanger, Andrew D. Randall

**Affiliations:** a Institute of Clinical and Biomedical Sciences, University of Exeter Medical School, University of Exeter, Exeter, UK; b King's College London, Institute of Psychiatry, Psychology & Neuroscience, Maurice Wohl Clinical Neuroscience Institute, London, UK

**Keywords:** Membrane electrical properties, Neuronal excitability, Synaptic plasticity, Tauopathy, Dementia, Alzheimer's disease, Progressive supranuclear palsy, Hippocampus

## Abstract

The accumulation of cleaved tau fragments in the brain is associated with several tauopathies. For this reason, we recently developed a transgenic mouse that selectively accumulates a C-Terminal 35 kDa human tau fragment (Tau35). These animals develop progressive motor and spatial memory impairment, paralleled by increased hippocampal glycogen synthase kinase 3β activity. In this neurophysiological study, we focused on the CA1 subfield of the hippocampus, a brain area involved in memory encoding. The accumulation of Tau35 results in a significant increase of short-term facilitation of the synaptic response in the theta frequency range (10 Hz), without affecting basal synaptic transmission and long-term synaptic plasticity. Tau35 expression also alters the intrinsic excitability of CA1 pyramidal neurons. Thus, Tau35 presence is associated with increased and decreased excitability at hyperpolarized and depolarized potentials, respectively. These observations are paralleled by a hyperpolarization of the voltage-sensitivity of noninactivating K^+^ currents. Further investigation is needed to assess the causal link between such functional alterations and the cognitive and motor impairments previously observed in this model.

## 1 Introduction

A range of neurodegenerative tauopathies in humans are characterized by the progressive accumulation of pathogenic tau aggregates within the central nervous system (CNS). These conditions are associated with diverse clinical outcomes, which may consist of both cognitive and motor impairments, as extensively reviewed (Goedert and Spillantini, 2011, Lee et al., 2001, Spillantini and Goedert, 2013). Alzheimer's disease represents the most common form of tauopathy. Its pathological outcomes coexist with, and may be a consequence of, amyloidopathy (Hardy and Allsop, 1991, Hardy and Higgins, 1992). Neurodegenerative diseases that are purely associated with the progressive accumulation of tau protein species, include frontotemporal dementia (FTD), progressive supranuclear palsy (PSP), and corticobasal degeneration (CBD). PSP is characterized histopathologically by neurodegeneration of midbrain and brainstem structures, including hypothalamus and basal ganglia (Pollock et al., 1986) and clinically by postural instability (Steele et al., 1964).

In tauopathy, the tau protein can be subjected to a range of disease-associated posttranslational modifications, the most explored one being its phosphorylation. The altered phosphorylation of tau protein leads to the formation of neurofibrillary tangles, and the occlusion of its ability to interact with microtubules; hyperphosphorylated tau is associated with all the previously mentioned neurodegenerative conditions (Spillantini and Goedert, 2013). Ubiquitination and tau truncation are also characteristic modifications observed in human tauopathies (Martin et al., 2011).

The majority of tauopathies are sporadic, and some lines of evidence suggest that the accumulation of tau fragments plays a part in the pathogenesis of some or all of these conditions. For instance, we previously identified a N-terminally truncated form of tau in human brain that is highly phosphorylated and associated with the development of 4-repeat forms of human tauopathy, such as PSP (Guo et al., 2017, Wray et al., 2008). This work on human disease tissues was followed-up by the production of a novel mouse model in which the brain-derived, C-terminal 35 kDa tau fragment (Tau35) was expressed under the control of the human tau promoter (Bondulich et al., 2016). The expression of the construct was introduced into the mouse hypoxanthine phosphoribosyl transferase (*Hprt*) locus (Bronson et al., 1996), guaranteeing single copy targeted integration. This both eliminates potential gene disruption around the insertion site and avoids artifacts due to tau overexpression. Correspondingly, in this animal, the Tau35 transgene was only expressed at a low level, accounting for less than 10% of the level of endogenous mouse tau.

Tau35 mice progressively develop a number of key aspects of human tauopathies. These include the appearance of aggregated and abnormally phosphorylated tau, increased glycogen synthase kinase 3β (GSK3β) activity, deficits in cognitive and motor function, premature death, dysfunction associated with autophagy and lysosomal processing, and a decline in synaptic protein levels. Cognitive decline was revealed in Tau35 mice by an age-dependent impairment in their performance in the Morris water maze, a well-validated hippocampal-dependent spatial memory task (Bondulich et al., 2016).

This work examines the neurophysiological function of the Tau35 mouse in the CA1 subfield of the hippocampus, an area with a well-established role in spatial navigation and memory (Chersi and Burgess, 2015). The experiments were all performed on mice aged 14–18 months. This is an age beyond which water maze learning is eliminated and is the age point at which biochemical and immunohistochemical measurements revealed a number of key alterations to hippocampal neurochemistry. These include tangle-like structures reactive for a broad range of tau antibodies, changes to tau phosphorylation, increased GSK-3β activity, altered levels of synaptic proteins, and modifications indicative of altered autophagic processing (Bondulich et al., 2016). Our experiments reveal that, as seen in other models of tauopathy (Booth et al., 2016b), long-term synaptic plasticity is unaltered in the Schaffer collateral commissural pathway. However, we did observe genotype-associated changes to short-term synaptic plasticity and intrinsic excitability properties of CA1 pyramidal neurons that may contribute to the loss of spatial memory these mice exhibit.

## 2 Materials and methods

### 2.1 Experimental animals

**Fig. 1:**
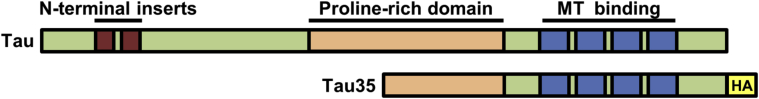
Schematic representation of the Tau35 fragment in comparison to full-length human tau. Tau is alternatively spliced, and the largest CNS isoform of human tau comprises 2 N-terminal inserts, followed by a proline-rich domain and 4 microtubule binding repeats (2N4R tau, 441 amino acids). The Tau35 sequence includes residues 187-441 of 2N4R human tau fused at the C-terminus to a hemagglutinin tag (HA). Abbreviations: CNS, central nervous system; Tau35, C-Terminal 35 kDa human tau fragment.

In all experiments, animals expressing the Tau35 transgene were compared against age-matched wild-type littermate controls (36 animals; 17 WT, 19 TG). Fig. 1 shows a schematic representation of the Tau35 fragment compared to human tau; this is the product of the expression of the Tau35 genetic construct described in our previous work (Bondulich et al., 2016). Animals were bred at King's College London and arrived in a single shipment to the University of Exeter Medical School prior to the commencement of the study. They were group housed, allowed food and water ad libitum and maintained on a 12:12 hours light/dark cycle. This study was carried out in accordance with UK Home Office Guidelines and the King's College London and University of Exeter Animal Welfare Ethical Review Board.

### 2.2 Preparation of brain slices

Animals were sacrificed using cervical dislocation in accordance with Schedule 1 of the Animals (Scientific Procedures) Act (1986). The brain was rapidly removed and transferred to an ice cold cutting solution consisting of (in mM): 189 sucrose, 10 D-glucose, 26 NaHCO_3_, 3 KCl, 5 MgSO_4_(7H_2_O), 0.1 CaCl_2_, and 1.25 NaH_2_PO_4_. About 300-μm coronal sections were cut using a Leica VT1200 microtome and immediately transferred to a holding chamber containing artificial cerebrospinal fluid (aCSF) continuously bubbled with carbogen. The composition of the aCSF was as follows (in mM): 124 NaCl, 3 KCl, 24 NaHCO_3_, 2 CaCl_2_, 1.25 NaH_2_PO_4_, 1 MgSO_4_, and 10 D-glucose. The slices were then allowed to recover for 30 minutes at 37 °C and subsequently at room temperature for at least 1 hour before transfer into a recording chamber. Field potential and whole-cell recording experiments were carried out on different brain slices.

### 2.3 Whole-cell patch-clamp recordings

Slices were transferred to a recording chamber where they were submerged in carbogen-equilibrated aCSF and maintained at a temperature between 33 °C–34 °C. The recording chamber was secured on the stage of an Olympus BX51 upright microscope and individual CA1 pyramidal neurons were visualized using infrared differential interference contrast optics. Borosilicate glass microelectrodes with a resistance ranging from 3–5 MΩ were pulled, fire-polished, and filled with a K-gluconate–based internal solution consisting of (in mM): 135 K-gluconate, 5 NaCl, 10 HEPES free acid, 0.2 EGTA, 0.3 Na-GTP, 4 Mg-ATP, and 13.4 Biocytin (pH 7.3, 280–290 mOsm). Following entry into whole-cell configuration, a junction potential error of 15 mV arose due to the pairing of the pipette solution with the aCSF, which was arithmetically corrected for during analysis. Signals were recorded using a Multiclamp 700B amplifier, digitized using a Digidata 1440, and stored for future analysis using pClamp 10 software.

All recordings were made from a defined prestimulus membrane potential set by injecting a continuous flow of bias current through the recording electrode. This facilitated the analysis of passive neuronal properties and action potential (AP) generation from prestimulus membrane potentials (V_m_) of both −80 and −74 mV. To measure neuronal passive membrane properties, a 500 ms, −100 pA hyperpolarizing current step was injected across the membrane from each V_m_. The subsequent voltage deflection at the steady state of the hyperpolarization was used to calculate the input resistance (R_in_) of the membrane using Ohm's law (V = IR). The extrapolation of a single exponential curve at an infinite time, fitted to the membrane charging response between 10% and 95% of the peak amplitude, was used to calculate the membrane time constant (τ). An approximation of capacitance was measured as the ratio between the τ and R_in_. Sag, measured as the difference between the negative peak and the steady state hyperpolarization, was expressed as a percentage of the peak hyperpolarization in response to a 500 ms, −100 pA hyperpolarizing current injection.

Standard “Zap” protocols were used to measure subthreshold membrane resonance properties, as previously described (Hu et al., 2002, Tamagnini et al., 2014). Briefly, the ratio of the fast Fourier transform of the voltage response versus the current injection was calculated as a measure of the impedance profile of the pyramidal neurons (Z = V_fft_/I_fft_). Subsequently, the impedance versus frequency profile was smoothed with a 35 point moving average function (sZ). Cell-to-cell maximal impedance (Z max, MΩ), frequency at which this maximal impedance occurred (Peak frequency, Hz), quality factor of the resonator (Q), and area under the curve (AUC, sZ*Fr) were quantified to facilitate comparisons between the genotypes.

To quantify neuronal excitability, a series of incremental 500 ms depolarizing square current injections, ranging from 50–300 pA, were injected across the membrane. The number of AP generated for each current injection was used as a measure of excitability. The pattern of neuronal firing was also examined by plotting the instantaneous frequency between APs versus the interval number. The first AP fired in response to a 300 pA depolarizing current step was used to compare AP waveforms between the genotypes. AP threshold was quantified, defined as the voltage at which the rate of rise (dV/dt) surpassed 20 V*s^−1^. AP width was measured at −15 mV, whereas the AP rate of rise (RoR) was measured for each cell as the rate of rise of the membrane potential at −20 mV during the rising phase of the AP.

Short-term plasticity was measured by evoking a train of 6 excitatory postsynaptic potentials (EPSPs) at 1 Hz, 3 Hz, 10 Hz, and 30 Hz. Each individual EPSP was elicited by delivering a 0.1 ms current pulse through a tungsten bipolar stimulating electrode placed on axons of the Schaffer collateral pathway. Stimulus intensity was set to produce measurable EPSPs without reaching threshold for triggering an AP. To analyze the relative increase in EPSP amplitude during the pulse train each value was normalized to the amplitude of the EPSP evoked by the first stimulus in the train and the area under curve was calculated for each cell: for each stimulation frequency, data were compared between genotypes.

For voltage clamp experiments, outside-out, somatic, nucleated macropatches were excised as previously described (Brown et al., 2011). Pipette capacitance was neutralized, and the series resistance was compensated for (10%–80% correction). Voltage-clamp recordings were made for the quantitative evaluation of outward-going plateau voltage-gated K^+^ currents, by applying 30-ms voltage steps, growing in 10-mV increments, and starting from a holding voltage of −90 mV. Each recorded current amplitude was normalized to the membrane capacitance of the macropatch to account for differences in current amplitude arising from different sizes of the macropatch. The specific current (pA/pF) was used to calculate the specific conductance to K^+^ ions based on a Nernstian equilibrium potential of −100 mV.

### 2.4 Field excitatory postsynaptic potentials

Following the initial postslicing recovery period, the CA3 region of hippocampal slices was removed using a scalpel cut. Slices were then transferred to a recording chamber in which they were submerged in continually perfused aCSF pre-equilibrated with carbogen and maintained at a temperature between 32 °C–33 °C. Field excitatory postsynaptic potentials (fEPSPs) were elicited by delivering a short pulse of electrical current (0.1 ms) through tungsten bipolar stimulating electrodes. These stimulating electrodes were placed such as to stimulate axons of the Schaffer Collateral (SC) or temporoammonic (TA) pathways. Borosilicate glass recording microelectrodes with a resistance ranging from 3–5 MΩ were pulled and filled with aCSF. Recorded signals were collected with an Axoclamp 2B amplifier, digitized with a Digidata 1322A, and stored for future analysis using pClamp 10 software.

For the majority of input-output curves and long-term potentiation (LTP) experiments, the SC and TA pathways were stimulated alternatively every 15 seconds; however, in some cases, experiments focused on an individual pathway. Input-output curves were constructed by incrementally increasing the current passing through the stimulating electrode and recording the evoked response (25–300 μA in 25 μA steps for the SC pathway, 125–1500 μA in 125 μA steps for the TA pathway). Following this, the threshold stimulation intensity required to elicit a visually detectable fEPSP was found. The stimulus intensity was then set at 3 times the threshold level for the remainder of the recording.

After a period of at least 20 minutes recording baseline responses at low frequency, induction of LTP was attempted in one pathway using a thetaburst stimulation protocol. This consisted of 5 bursts of 10 stimuli at 100 Hz applied with an interburst interval of 200 ms. This was repeated 4 times with an inter-repeat interval of 20 seconds. The fEPSPs were then followed for 1 hour before the thetaburst stimulation protocol was delivered to the other pathway and fEPSPs were followed for another hour. The order in which the pathways were stimulated was alternated between experiments.

### 2.5 Data analysis

Data were analyzed using a range of custom written MATLAB scripts, pClamp, and Origin 9.1 analysis tools. The approaches used for the analysis of intrinsic properties are described in the study by Tamagnini et al. (2014). Statistical assessments of differences between genotypes were made using unpaired 2-tailed Student *t* tests (with or without Bonferroni correction -bc- for multiple comparisons) and 2-way analysis of variance (ANOVA) as appropriate; non-parametric Mann-Whitney *U* test was used for normalized data. Figures were prepared with Origin 9.1. Data are shown as mean ± standard error of the mean (SEM) values.

## 3 Results

High levels of 35 kDa cleaved tau protein (Tau35) have been found in the brain of patients affected by PSP (Wray et al., 2008). By performing electrophysiological recordings in the CA1 subfield of the dorsal hippocampus of 14- to 18-month old Tau35 mice and age-matched littermate controls, our study aimed to characterize the functional neurophysiological consequences of chronic exposure to C-terminal 35 kDa fragments of tau protein. First, we used extracellular field potential recordings to examine synaptic transmission and long-term plasticity at excitatory synapses in the CA1 subfield of the hippocampus. Historically, this has unquestionably been the most commonly made assessment of neurophysiological function in rodent models of human dementia (Randall et al., 2010). To parallel other recent studies in our laboratory (Booth et al., 2014, Booth et al., 2016b), we looked at both Schaffer collateral (SC)-CA1 and temporoammonic (TA) synaptic function. Contemporaneously, but on separate slices, we employed whole-cell patch-clamp recordings from single CA1 pyramidal cells to assess both short-term synaptic plasticity on SC-CA1 synapses and the intrinsic excitability membrane properties of CA1 excitatory pyramidal neurons.

**Fig. 2:**
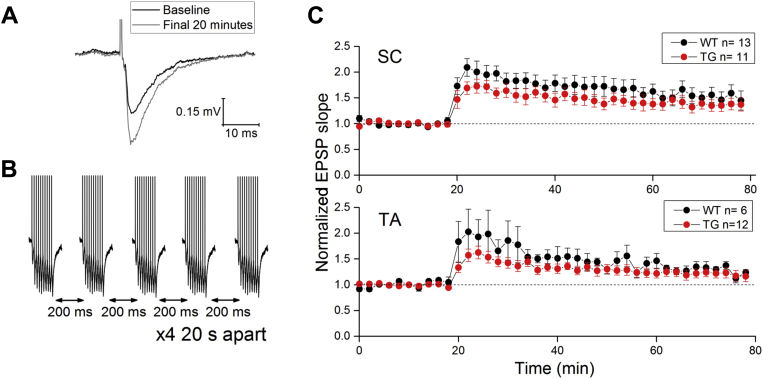
Long-term potentiation (LTP) in CA1 of hippocampal slices prepared from Tau35 mice is normal. (A) Average trace of an evoked fEPSP during the baseline (black) and final 20 minutes (gray) of a single SC LTP experiment. (B) Sample traces of the theta burst induction protocol were used to induce LTP. (C) Extracellular recordings of evoked fEPSPs in hippocampal CA1 do not show differences in LTP induction between Tau35 and age-matched WT littermate controls. We tested 2 different pathways to CA1: the schaffer collateral (SC) pathway (top panel) and the temporoammonic (TA) pathway (bottom panel). Abbreviations: fEPSP, field excitatory postsynaptic potential; Tau35, C-Terminal 35 kDa human tau fragment.

Extracellular recordings of evoked field EPSPs revealed no difference in basal synaptic transmission in either pathway (data not shown; CA1-SC–2-way repeated measures [RM] ANOVA, source of variability = genotype, F = 0.95, *p* = 0.35; TA-CA1–2-way RM ANOVA, source of variability = genotype, F = 1.45, *p* = 0.26). We also observed no difference in the ability to induce LTP in either SC-CA1 or TA-CA1 synapses (Fig. 2; SC-CA1–Mann-Whitney *U* Test, source of variability = genotype; *p* = 0.4; TA-CA1–Mann-Whitney *U* Test, source of variability = genotype, *p* = 0.46) in Tau35 mice when compared with their WT littermate controls.

**Fig. 3:**
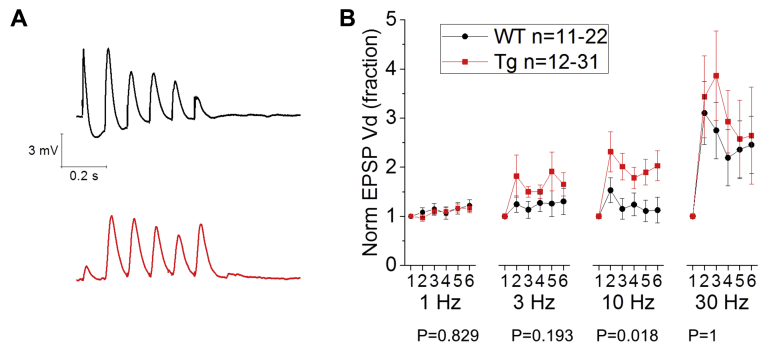
Increased facilitation of the excitatory synaptic transmission in the SC-CA1 pathway at theta range (10 Hz) stimulation frequencies in Tau35 CA1-PCs. (A) Example traces showing 6 evoked EPSPs from 2 patch-clamp, current-clamp recordings in CA1-PCs from WT (top-black) and Tau35 (bottom-red) mice; in this case, facilitation was obtained by delivering the stimuli on the SC pathway at 10 Hz (theta range). (B) Each cell received a complete set of stimulus frequencies: 1 Hz, 3 Hz, 10 Hz (theta range), and 30 Hz (low-gamma range). Although no differences between genotypes were observed at 1 Hz, 3 Hz, and 30 Hz (at this latter frequency both genotypes showed sustained facilitation), at 10 Hz, Tau35 CA1-PCs showed sustained facilitation along all of 6 pulses, whereas WT controls came back to baseline after the second pulse. Abbreviations: EPSPs, excitatory postsynaptic potentials; Tau35, C-Terminal 35 kDa human tau fragment; WT, wild-type mouse. (For interpretation of the references to color in this figure legend, the reader is referred to the Web version of this article.)

In addition, whole-cell patch-clamp recordings of EPSPs in CA1-PCs, evoked by the repeated stimulation of SC fibers (6 pulses delivered at 1 Hz, 3 Hz, 10 Hz, and 30 Hz), revealed an increase in short-term synaptic plasticity in Tau35 mice. This was apparent at a stimulus frequency of 10 Hz, a frequency within the theta band range but not at the 3 other stimulation frequencies tested (Fig. 3; Mann-Whitney *U* test, source of variability = genotype; 1 Hz, *p* = 0.829; 3 Hz, *p* = 0.193; 10 Hz, *p* = 0.018; and 30 Hz, *p* = 1).

**Fig. 4:**
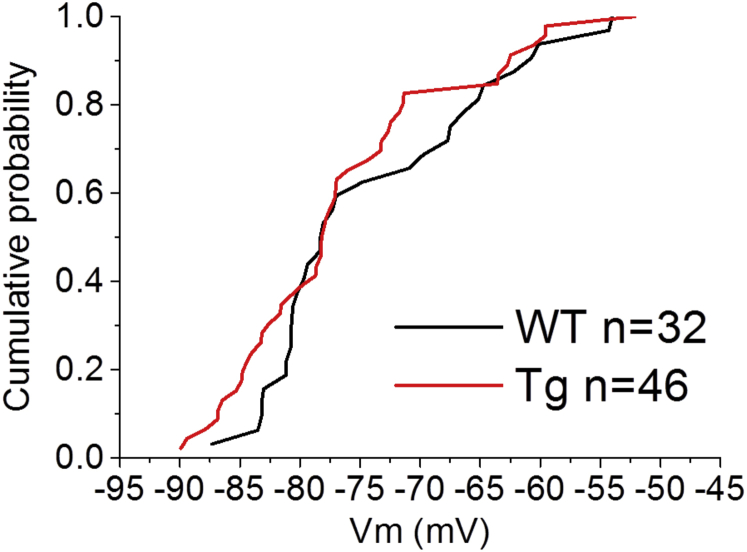
Tau35 CA1-PCs had similar RMPs to WT neurons. Cumulative probability curve showing cell-to-cell RMP probability in Tau35 and age-matched WT littermate controls. No differences were observed between genotypes (see Table 1 for mean ± standard error of the mean and *p*-values). Abbreviations: RMP, resting membrane potential; Tau35, C-Terminal 35 kDa human tau fragment; WT, wild-type mouse.

We, and others, have described differences in intrinsic excitability membrane properties of hippocampal neurons in a number of murine models of human dementia (Brown et al., 2011, Kerrigan et al., 2014, Siskova et al., 2014, Tamagnini et al., 2015a, Tamagnini et al., 2015b, Wykes et al., 2012), including models of FTD (Booth et al., 2016b). Consequently, we compared intrinsic excitability properties of Tau35 and WT control mice using whole-cell current-clamp recordings from CA1-PCs. No differences were observed in the resting membrane potential (RMP) (Fig. 4; Tau35, RMP = −76.6 mV ± 1.3 mV n = 47 and WT, RMP = −74.1 mV ± 1.6 mV n = 32, unpaired Student's *t* test *p* = 0.2). To avoid biases arising from cell-to-cell variability in RMP, all the other intrinsic excitability properties were measured from 2 fixed prestimulus V_m_, −80 mV and −74 mV, respectively. These prestimulus levels were established with a suitable level of bias current injection. We chose to record the intrinsic excitability properties from the 2 different prestimulus potentials as we wanted to gather information on the intrinsic excitability properties arising from a potential that was (1) close to the average value RMP (i.e., −74 mV) and (2) sufficiently hyperpolarized to measure subthreshold oscillatory properties of the cell (i.e., −80 mV).

**Fig. 5:**
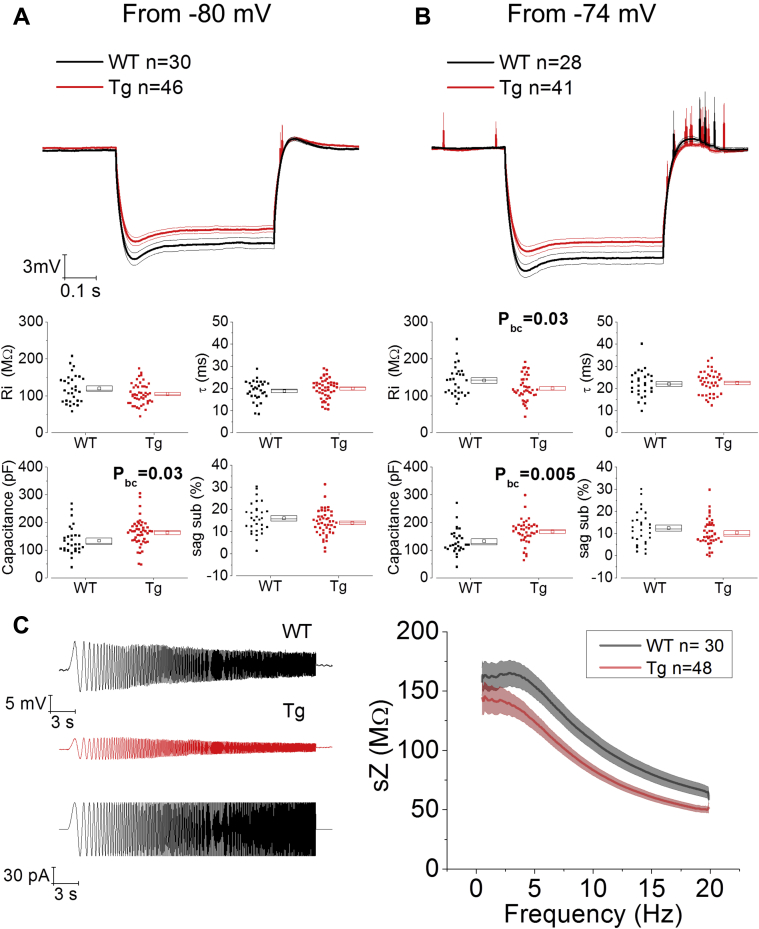
Tau35 CA1-PCs have altered passive membrane properties. (A) and (B): Passive membrane properties of CA1-PCs were tested in Tau35 and age-matched WT littermate controls with current-clamp whole-cell V_m_ recordings on the injection of a −100 pA, 500 ms current step at 2 different fixed prestimulus potentials, −80 mV (A) and −74 mV (B), obtained with a constant current injection. Top panels show the averaged ± standard error of the mean (SEM) V_m_ responses, whereas the bottom panels show scatter plots of cell-to-cell analysis of the tested passive properties: R_in_, τ, capacitance, and sag. The boxes on the right of each scatter distribution (in this and following figures) represent the mean (small square) median and SEM boundaries. Although capacitance was higher at both tested voltages, R_in_ was significantly reduced only when tested at V_m_ = −74 mV (unpaired Student's *t* test with Bonferroni correction: P_bc_). (C) Impedance was reduced in the resonance profile of Tau35 mice (right panel), obtained with the injection of a subthreshold sinusoidal oscillating current with a frequency linearly increasing between 0.5 Hz and 20 Hz (left panel, bottom trace); the top traces in the left panel are representative examples, for each genotype, of the V_m_ oscillating in response to the sinusoidal current injection. Abbreviation: Tau35, C-Terminal 35 kDa human tau fragment.

The hyperpolarization response resulting from the injection of a −100 pA, 500 ms square current step, allowed us to measure membrane passive properties, such as R_in_, τ, and capacitance of the membrane. This response also allowed us to measure the I_h_–dependent “sag”, we have found to be altered in rTg4510 mice, a transgenic model of progressive tauopathy (Booth et al., 2016b). Two-way RM ANOVA showed an overall effect of genotype (between subjects) and voltage (within subjects) but no interaction between genotype and voltage for R_in_ (genotype: F = 6.06, *p* = 0.016; voltage: F = 177.05, *p* < 0.0001; genotype*voltage: F = 0.933, *p* = 0.34). Unpaired Student *t* test with Bonferroni correction for multiple comparisons, revealed that R_in_ was not significantly different between genotypes when the prestimulus V_m_ was held at −80 mV (Fig. 5A, see bottom panels), but it was significantly lower in Tau35 mice when the prestimulus V_m_ was −74 mV (Fig. 5B, see bottom panels). In addition, capacitance was significantly higher in Tau35 mice at both tested prestimulus V_m_ (Fig. 5, bottom panels), but no effect of voltage nor interaction between genotype and voltage was observed (voltage: F = 0, *p* = 0.99; genotype*voltage: F = 0.510, *p* = 0.478). No differences were observed in other passive properties examined, such as sag and τ. The lack of change in τ reflects the balancing of the ∼20% increase in capacitance by a reciprocal decrease in R_in_ (although this was not statistically significant at −80 mV). See Table 1 for mean ± SEM, F, and *p*-values.

**Table 1:** Passive membrane properties measured in CA1-PCs of Tau35 and WT mice at 2 prestimulus set potentials: V_m_ = −80 mV and V_m_ = −74 mV

Property	WT (n = 28–32)		Tau35 (n = 40–46)		P_bc_	2-Way RM ANOVA (genotype)
	Mean	SEM	Mean	SEM		
R_i_ (MΩ) at V_m_ = −80 mV	119.92	6.99	105.38	4.25	0.12	F = 6.06 *p* = 0.016
R_i_ (MΩ) at V_m_ = −74 mV	141.74	7.65	120.10	5.12	0.03	
sag (%) at V_m_ = −80 mV	16.01	1.24	13.94	0.85	0.32	F = 1.99 *p* = 0.163
sag (%) at V_m_ = −74 mV	12.50	1.43	10.43	0.97	0.43	
τ (ms) at V_m_ = −80 mV	18.95	0.84	19.98	0.66	0.67	F = 0.40 *p* = 0.529
τ (ms) at V_m_ = −74 mV	22.06	1.14	22.50	0.83	1	
Cap (pF) at V_m_ = −80 mV	134.22	9.30	163.71	7.46	0.03	F = 10.21 *p* = 0.002
Cap (pF) at V_m_ = −74 mV	132.52	8.68	167.08	7.05	0.005	

Mean ± standard error of the mean and *p*-values (unpaired Student *t* test with Bonferroni correction -P_bc_-for multiple comparisons) are shown for each.

Key: Tau35, C-Terminal 35 kDa human tau fragment; WT, wild-type mouse.

If an oscillating current of linearly increasing frequency and of subthreshold intensity is injected, the oscillatory impedance properties of the cell can be explored. Our data showed that the cell impedance (Z) was lower across the whole frequency spectrum, as revealed by cell-to-cell analysis of AUC in frequency versus impedance (Fig. 5C, right panel; Tau35, AUC = 1747 Hz*MΩ ± 103 Hz*MΩ, n = 48; WT, AUC = 2191 Hz*MΩ ± 153 Hz*MΩ, n = 30, unpaired Student *t* test *p* = 0.01). This finding is in line with the lower R_in_ in this group (Fig. 5 and Table 1). However, no significant changes were observed in cell-to-cell analysis of peak Z, peak frequency, and the quality factor of the resonator (Q), as summarized in Table 2 (showing mean ± SEM and *p*-values).

**Table 2:** Subthreshold resonance membrane properties measured in CA1-PCs of Tau35 and WT mice at 2 prestimulus set potentials: V_m_ = −80 mV and V_m_ = −74 mV

Property	WT n = 30		Tau35 n = 48		*p*-value
	Mean	SEM	Mean	SEM	
Peak Fr (Hz)	2.8	0.2	2.5	0.2	0.3
Peak Z (MΩ)	169.8	11.4	148.7	12.0	0.2
Q	1.07	0.01	1.05	0.01	0.4
AUC (MΩ*Hz)	1747	103	2191	153	0.01

Mean ± SEM and *p*-values (unpaired Student's *t* test) are shown for each.

Key: Tau35, C-Terminal 35 kDa human tau fragment; WT, wild-type mouse.

**Fig. 6:**
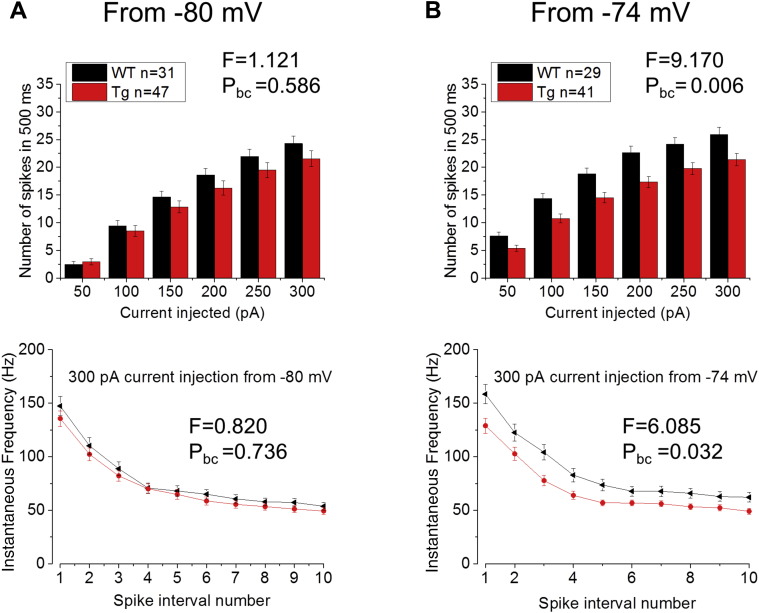
Tau35 CA1-PCs are hypoexcitable at depolarized but not hyperpolarized potentials. The average (top panels) and instantaneous (bottom panels; only +300 pA step is shown) firing frequencies of CA1-PCs were tested in Tau35 and age-matched WT littermate controls with current-clamp whole-cell V_m_ recordings on the injection of 500 ms, +50 pA to +300 pA. Although no differences were observed between genotypes at V_m_ = −80 mV (A), a significant reduction of both average and instantaneous firing frequencies was observed in Tau35 mice at V_m_ = −74 mV (B); such reduction in firing rate at more depolarized potentials is probably a reflection of the respective reduction in R_in_, as described in Fig. 5A and B. Abbreviation: Tau35, C-Terminal 35 kDa human tau fragment.

Suprathreshold firing properties were explored using 500 ms duration step injections of depolarizing current. These ranged in amplitude from −50 pA to 300 pA in 50 pA increments. As for the determination of passive properties, these were applied from a prestimulus membrane potential of both −80 mV and −74 mV. With a prestimulus potential of −80 mV, no significant differences between genotypes were observed in the total spike output (Fig. 6A, top panel; 2-way RM ANOVA, source of variability = genotype, F = 1.121, P_bc_ = 0.586) or dynamics of spike firing. The latter is illustrated for a 300 pA stimulus by the bottom panel of Fig. 6A (bottom panel; 2-way RM ANOVA, source of variability = genotype, F = 0.820, P_bc_ = 0.736). In contrast, when measured from a prestimulus membrane potential of −74 mV, the total spike output was significantly lower in Tau35 mice compared to age-matched WT controls (Fig. 6B, top panel; 2-way RM ANOVA, source of variability = genotype, F = 9.170, P_bc_ = 0.006). This was also reflected in the temporal dynamics of firing as illustrated for a 300 pA stimulus in the bottom panel of the figure (Fig. 6B 2-way RM ANOVA, source of variability = genotype, F = 6.085, P_bc_ = 0.032). These results show a reduction of excitability of hippocampal CA1-PCs in Tau35 mice, at slightly more depolarized RMPs.

**Fig. 7:**
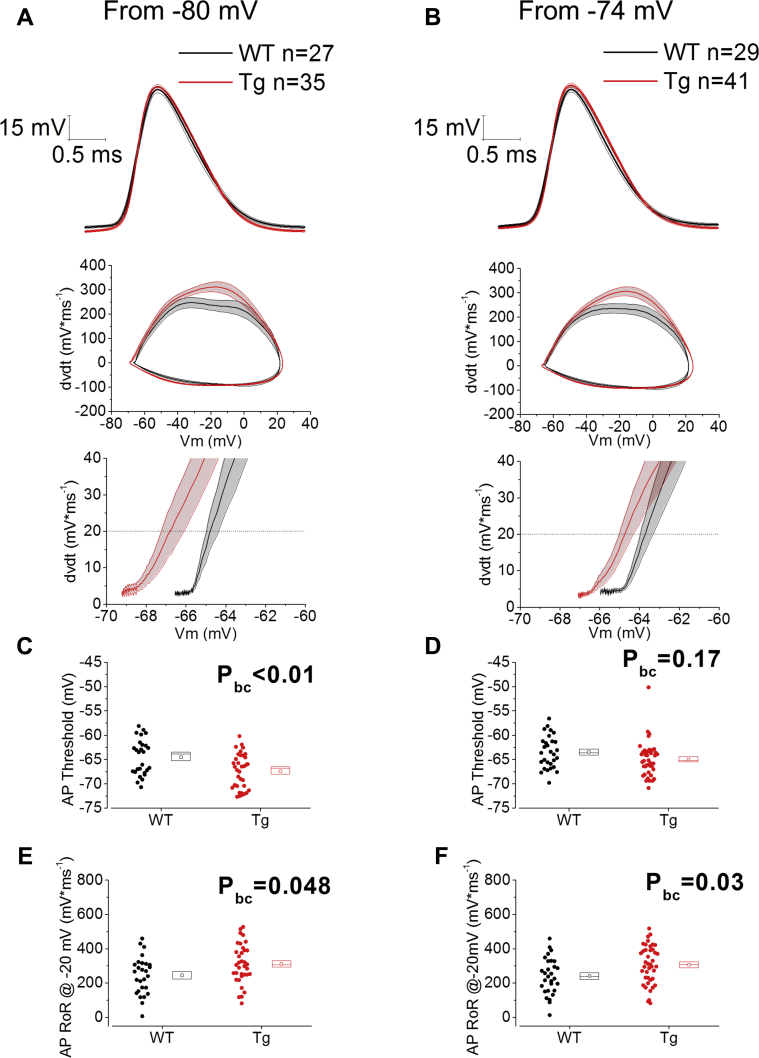
Tau35 CA1-PCs have altered action potential kinetics. (A) and (B): Top panels show the averaged ± standard error of the mean V_m_ responses corresponding to the first AP fired on the injection of a 500 ms, +300 pA recorded from CA1-PCs in Tau35 and age-matched WT littermate controls from a prestimulus potential of −80 mV (A) or −74 mV (B); middle panels show the corresponding phase plots of the first V_m_ derivative in time versus V_m_: these show a visible difference in the rate of rise around a V_m_ = −20 mV and hyperpolarization of the AP threshold, more clearly visible in the corresponding expanded phase plot in the bottom panel. (C–F). Cell-to-cell analysis of the AP waveform properties, revealed a significant increase of AP rate of rise at both −80 mv (E) and −74 mV (F) prestimulus V_m_, while the AP threshold resulted significantly hyperpolarized only at V_m_ = −80 mV (C) but not at V_m_ = −74 mV (D); this could be due to a voltage-dependent dampening effect, the same one that provoked voltage-dependent decrease in R_in_ and firing rate. Abbreviations: AP, action potential; Tau35, C-Terminal 35 kDa human tau fragment; WT, wild-type mouse.

As well as characterizing the extent and dynamics of depolarization-induced AP firing, we also investigated the genotype-dependence of AP waveform as we (Brown et al., 2011, Kerrigan et al., 2014, Tamagnini et al., 2015a, Tamagnini et al., 2015b) and others (Wykes et al., 2012) have seen this change in other rodents exhibiting dementia-associated pathology. In this regard, we observed an overall effect (2-way RM ANOVA) of genotype on the AP threshold, but no effect of voltage nor interaction between genotype and voltage (genotype: F = 4.923, *p* = 0.031; voltage: F = 0.004, *p* = 0.948; genotype*voltage: F = 1.816, *p* = 0.183). Multiple comparisons between genotypes at each voltage revealed a 3-mV hyperpolarization of the AP threshold in Tau35 mice versus age-matched WT littermate controls for recordings made from a prestimulus holding V_m_ = −80 mV (Fig. 7C; unpaired *t* test with Bonferroni correction for multiple comparisons, P_bc_ = 0.006). Although the AP threshold was 1.5 mV more hyperpolarized in Tau35 mice when current stimuli were delivered to cells at −74 mV, this was not a significant difference (Fig. 7D; unpaired *t* test with Bonferroni correction for multiple comparisons, *p* = 0.17). In addition, the rate of rise of the AP measured in each cell at −20 mV was significantly faster in Tau35 mice and slower (as an overall effect of voltage across genotypes) at more depolarized potentials (Fig. 7E and F; 2-way RM ANOVA for genotype: F = 5.661, *p* = 0.021; voltage: F = 4.197, *p* = 0.045; genotype*voltage: F = 0.124, *p* = 0.726). Unpaired Student *t* tests between genotypes with Bonferroni correction for multiple comparisons at both prestimulus potentials tested, revealed that the AP rate of rise was higher in Tau35 mice at both tested potentials (P_bc_ = 0.048 at −80 mV and *p* = 0.03 at −74 mV, respectively). AP width and AP peak were not significantly different between genotypes. For a summary of AP waveform properties see Table 3. This observation allows us to conclude that CA1-PCs from Tau35 are hyperexcitable, and this effect might be more pronounced at more polarized potentials. Series resistance (R_s_) was compensated for in each recording; R_s_ was not different between genotypes (WT R_s_ = 13.5 ± 0.3; Tau35 R_s_ = 13.5 ± 0.4, unpaired Student *t* test *p* = 0.97).

**Table 3:** AP waveform properties measured in CA1-PCs of Tau35 and WT mice at 2 prestimulus set potentials: V_m_ = −80 mV and V_m_ = −74 mV. Mean ± SEM and *p*-values (2-way ANOVA and unpaired Student *t* test with Bonferroni correction -P_bc_-for multiple comparisons) are shown for each

Property	WT (n = 26–29)		Tau35 (n = 30–41)		P_bc_	2-Way RM ANOVA (genotype)
	Mean	SEM	Mean	SEM		
Width (ms) at V_m_ = −80 mV	0.80	0.04	0.82	0.02	1	F = 0.698 *p* = 0.407
Width (ms) at V_m_ = −74 mV	0.80	0.04	0.84	0.02	0.6	
Peak (mV) at V_m_ = −80 mV	21.59	2.10	23.24	1.53	1	F = 0.690 *p* = 0.410
Peak (mV) at V_m_ = −74 mV	21.60	1.71	24.23	1.45	0.5	
Thr (mV) at V_m_ = −80 mV	-64.53	0.71	-67.38	0.61	0.006	F = 4.923 *p* = 0.031
Thr (mV) at V_m_ = −74 mV	-63.46	0.62	-64.94	0.56	0.2	
RoR (V/s) at V_m_ = −80 mV	245.02	21.01	311.59	19.35	0.048	F = 5.661 *p* = 0.021
RoR (V/s) at V_m_ = −74 mV	240.69	19.63	306.81	17.54	0.03	

Key: AP, action potential; RoR, rate of rise; Tau35, C-Terminal 35 kDa human tau fragment; WT, wild-type mouse.

**Fig. 8:**
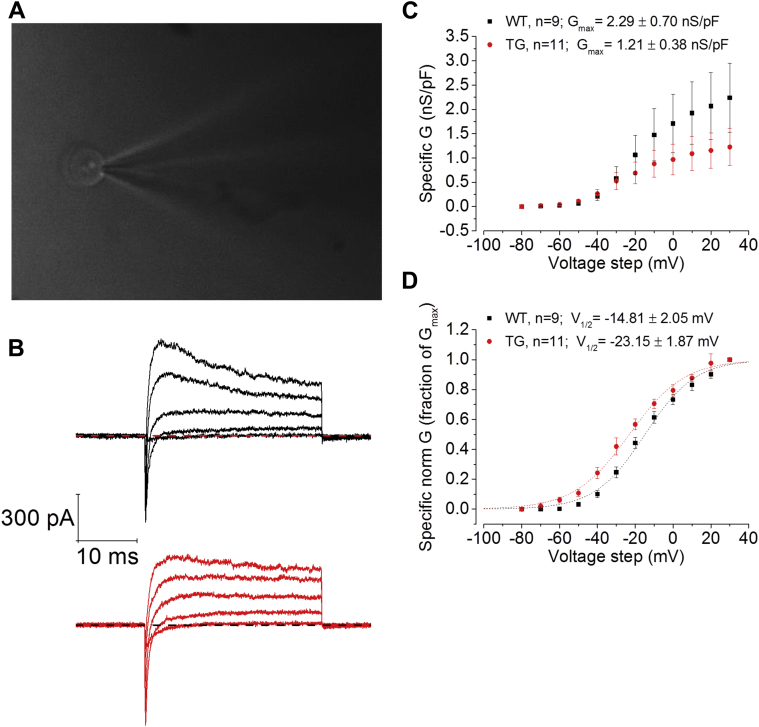
Activation of somatic noninactivating K^+^ currents occurs at more negative potentials in Tau35 CA1-PCs. (A) Photograph of an outside-out nucleated somatic macropatch, excised from a CA1-PC of a Tau35 mouse to measure the biophysical properties of voltage-gated noninactivating outward currents (I_K_). (B) Example current traces evoked by 30 ms, +40/+60/+80/+100/+120 mV voltage steps, from a prestimulus voltage clamped at −90 mV. After fast inactivating inward and outward currents, the I_K_ persisted until the end of the voltage step stimulation. (C) Sigmoidal increase of I_K_ specific conductance from a baseline V_m_ = −90 mV on 10-mV voltage steps. Analysis of I_K_ biophysical properties was performed by calculating the maximal conductance (G_max_) and voltage-sensitivity (V_1/2_) with cell-to-cell Boltzman fits. No effect of the genotype was observed on I_K_'s G_max_; however, a significant hyperpolarization of the V_1/2_ was observed in Tau35 CA1-PCs. This can be more clearly appreciated in (D), which shows the IV curve normalized versus the measured G maximal value for each cell. (C) and (D) show mean ± standard error of the mean values while the dashed lines represent the sigmoidal Boltzman fit for the mean values. Abbreviation: Tau35, C-Terminal 35 kDa human tau fragment.

The changes to intrinsic excitability properties outlined above likely have a significant component of their basis in the underlying ion channel complement of CA1 pyramidal cells (CA1-PCs). In the past, we have studied changes of this nature in dementia models using voltage clamp methods to characterize voltage-gated current components (Brown et al., 2011). Notably, due to the complex geometry and size of pyramidal neurons, and the large currents they express, using conventional whole-cell methods for such recordings can have limited utility. To circumvent these issues, we employed, as in our previous work (Brown et al., 2011), nucleated somatic macropatches to examine voltage-gated channels. An example of an outside-out, somatic macropatch excised from a CA1 cell in this study is shown in the top panel of Fig. 8A; example voltage-gated outward K^+^ currents are shown below (Fig. 8B). Pooled data plotting the activated K^+^ conductance versus voltage relationships from 9 WT and 10 Tau35 macropatches are shown in Fig. 8B. No effect of genotype was observed on G_max_ (Fig. 8C; WT n = 9, G_max_ = 2.3 ± 0.7 nS/pF vs Tau35 n = 11, G_max_ = 1.2 ± 0.4 nS/pF; *p* = 0.2), possibly due to the considerable cell-to-cell variability in the populations of macropatches from both genotypes. We did observe, however, a striking and significant shift in the voltage dependence of K^+^ current activation, which is better illustrated by the peak-normalized conductance-voltage plots shown in Fig. 8D. These highlight the 8 mV more negative activation curve seen in Tau35 mice (Fig. 8C; WT n = 9, V_1/2_ = −14.81 ± 2.05 mV vs. Tau35 n = 11, V_1/2_ = −23.15 ± 1.87 mV; *p* = 0.0076). The Boltzmann slope factor of the current activation was not affected by genotype. These results underlie and parallel the reduction of excitability observed in Tau35 mice at more depolarized potentials.

## 4 Discussion

Tauopathies are neurodegenerative disorders characterized by the progressive accumulation of neurotoxic hyperphosphorylated tau species within the CNS. Tauopathy is the major hallmark of several neurodegenerative diseases that are predominantly characterized by cognitive decline and dementia, for example Alzheimer's disease and Pick's disease. Tauopathy is also a cardinal feature of certain diseases where cognitive decline is accompanied by progressive development of motor deficits, such as PSP and CBD (Goedert and Spillantini, 2011, Iqbal et al., 2016, Lee et al., 2001, Lopez Gonzalez et al., 2016, Spillantini and Goedert, 2013, Wray et al., 2008). Even if some tauopathies are genetic disorders, most of them are sporadic events, depending on both genetic (Mitsui and Tsuji, 2014) and environmental risk factors. It has been shown, for instance, that a highly phosphorylated 35 KDa fragment of tau protein in human brain is associated with tauopathies, such as PSP (Wray et al., 2008). Tau35 transgenic mice were developed to model the accumulation of the 35 KDa C-term fragment of human tau, and the first characterization of these mice was recently published. It described neurological symptoms relating to both progressive motor and cognitive decline paralleling the progressive tauopathy. In addition, it showed the progressive accumulation of hyperphosphorylated pathogenic tau forms associated to the Tau35 genotype, paralleled by GSK-3β accumulation and autophagy impairment (Bondulich et al., 2016). In this respect, Tau35 mice can perhaps be regarded as a first model, expressing wild-type tau, of sporadic tauopathies such as PSP, FTD, and CBD.

In this study, we have made a first neurophysiological characterization of Tau35 mice. We chose to examine the CA1 area of the dorsal hippocampus as this has been widely studied in many dementia models, and it is pertinent to the spatial memory deficit reported in these animals using the Morris water maze (Bondulich et al., 2016). We examined the aspects of both synaptic function and single cell intrinsic excitability properties in a single study cohort of 14- to 18-month-old Tau35 and age-matched WT littermate controls.

Extracellular field recordings of EPSPs indicated that brain slices prepared from Tau35 mice exhibited normal theta burst–induced LTP in Schaffer collateral/CA1 synapses. LTP was also intact when synaptic inputs to the deep dendrites of CA1 cells such as the temporoammonic pathway were stimulated (Fig. 2). Although these outcomes may be surprising for some, LTP in various hippocampal pathways has been reported to be intact in many transgenic models of human dementia pathology, including both models of amyloidopathy (Brown et al., 2005, Fitzjohn et al., 2001, Hsia et al., 1999) and tauopathy (Booth et al., 2016b, Maeda et al., 2016, Randall et al., 2010, Scott et al., 2016).

When the short-term facilitation of Schaffer collateral EPSPs was tested with whole-cell current-clamp recordings of stimulus train-evoked EPSPs in CA1-PCs, a difference in short-term synaptic plasticity was seen. When EPSPs were driven with short 1 Hz trains, the responses from the 2 genotypes were almost identical with a 20% response facilitation developing over the 6 pulse train. With 3-Hz trains, Tau35 responses appeared to grow more, but this did not quite reach a significant level; however, a similar and statistically significant outcome was seen with 10-Hz synaptic activation. Thus, in Tau35 mice, EPSPs both initially grew more and maintained their increase throughout the train. This meant the overall depolarizing envelope of the EPSP was some 55% larger. Interestingly, the frequency at which EPSP facilitation is most striking, lies in the theta frequency range, paralleling the most prominent in vivo hippocampal network oscillation in awake behaving rodents, and one that is also impaired in other models of tauopathy (Booth et al., 2016b).

It is interesting to consider the potential underlying mechanism for the enhanced short-term plasticity at Schaffer collateral synapses in Tau35 mice. This could reflect a lower basal probability of release, and although extracellular recordings of input-output curves did not suggest this, these could be affected by other factors including an enhanced axonal excitability. This hypothesis is in line with the decrease in synapsin-1 we previously observed in Tau35 (Bondulich et al., 2016) where we speculated that such decrease might result in a selective loss of releasable vesicles but not synaptophysin-regulated endocytosis of synaptic vesicles. This result parallels the observation of reduced amplitude of 10 Hz evoked EPSPs in cultured rat hippocampal neurons and drosophila neuromuscular junctions, carrying mutations associated to familial FTD (i.e., P301L on tau) (Zhou et al., 2017). The authors hypothesized that this tauopathy-associated synaptic deficit is caused by the presynaptic interaction of the mutated tau N-terminus with neurotransmitter-carrying vesicles. We observed an opposite result in Tau35 mice CA1-PCs, where 10 Hz facilitation was in fact more prominent in Tau35 mice in comparison to their WT littermates: the Tau35 fragment is an N-terminus truncated, C-terminus peptide, and the increased synaptic facilitation we observed might be related to its accumulation. However, a more controlled and direct experimental assessment of release probability would certainly be a worthwhile endeavor. This could be achieved, for example, with either emptage optical quantal methods or by studying the rate of block of postsynaptic EPSPs with a non-competitive NMDA-R antagonist such as MK-801. The time-course of the effect during training, however, may point to an effect on presynaptic intracellular Ca^2+^ handling. A reduced ability to dynamically buffer presynaptic Ca^2+^ would likely produce an outcome akin to that reported in Fig. 3. In this respect, it would be interesting, as a future development of the present work, to use imaging to measure the kinetics of presynaptic AP induced Ca^2+^ transients in CA3 pyramidal cells.

If one embraces the well-established concept that an LTP-like process is pivotal to the establishment of hippocampal-dependent memory traces, one might ask how the normal LTP in Tau35 mice can be reconciled with the deficits these animals develop in a hippocampal-dependent learning task (Bondulich et al., 2016). A potential solution to this dichotomy lies in consideration of the differences between the nature of a typical LTP experiment and how synaptic plasticity is generated in vivo. In the experimental situation, a stimulating electrode is used to simultaneously activate many presynaptic fibers both during the plasticity-inducing conditioning stimulus (typically a 100 Hz train or theta burst stimulus) and in the pre- and post-conditioning test periods. In vivo, however, plasticity-inducing patterns of neural firing are not produced by stimulating electrodes, but they instead relate to the intrinsic properties of the communicating neurons and how these shape various aspects of on-going input to the cell. Consequently, disease-associated changes to intrinsic properties could reduce or eliminate the generation of particular patterns of firing, including those required to induce LTP-like synaptic plasticity. For this and other reasons, we believe it is a useful endeavor to establish how intrinsic neuronal properties are modified by pathologies, as we and others have done in this and a number of other recent investigations (Booth et al., 2016a, Booth et al., 2016b, Brown et al., 2011, Kerrigan et al., 2014, Tamagnini et al., 2015a, Tamagnini et al., 2015b, Wykes et al., 2012).

No differences in CA1-PC RMP were observed when comparing the genotypes. This agrees with our observations in another model of tauopathy (Booth et al., 2016a, Booth et al., 2016b) and our investigations of CA1-PC in a variety of other models relating to human dementia (Brown et al., 2011, Kerrigan et al., 2014, Tamagnini et al., 2015a, Tamagnini et al., 2015b). Although RMP was not altered, we did uncover changes to other intrinsic properties in experiments in which defined current pulses were injected into the cell via the recording electrode. These experiments were performed from 2 set prestimulus membrane potentials, −80 mV to match much of our previous work, and −74 mV, which was closer to the mean RMP in this study. The capacitance, calculated from the ratio of input resistance and membrane time constant, was increased at both V_m_ tested. Indeed, as this measure should predominantly arise from the surface area of the cell and the insulating properties of the lipid bilayer, it would be surprising if capacitances were found to be voltage-dependent. In line with this, the values determined at the 2 V_m_ within each genotype were within <2.5% of each other. The 20%–23% increase in capacitance is probably due to the CA1-PCs in Tau35 mice being larger. If the cells were a simple sphere, this would be expected to equate to about a 6% increase in diameter or 12%–13% increase in area, and although CA1-PC are geometrically much more complex, a careful quantitative analysis of morphology would seem warranted. Another less likely possibility is that the dielectric properties of the membrane have changed, something which could arise from a substantial change to the biochemical make-up of the lipid bilayer.

Membrane time constant, at around 20 ms, was very similar in Tau35 and WT mice, but R_in_ was lower in the former group (thus resulting in the calculated capacitance outcome as described previously). This lower R_in_ was only significantly different (by 15%) at −74 mV, whereas the 12% decrease observed at −80 mV was not significant. It is possible that the decrease in R_in_ represents a genomically mediated compensatory mechanism engaged by the neurons to maintain their membrane time constant in the face of an increased capacitance. Commensurate with the expected Ohmic consequences of a lower R_in_ (i.e., a smaller voltage change for any given current), we observed a reduced spike output and lower spiking rates in CA1-PC at −74 mV (Fig. 6). In fractional terms, these differences in spike output are greater with weaker current stimuli, which are more likely to have physiological pertinence. Thus, spike output is down by close to 50% when 50 pA is injected to cells at −74 mV, whereas the difference is only circa 20% with a 300 pA stimulus.

The observation of a genotype-related difference between measurements made from −80 mV and −74 mV points to the involvement of an ionic conductance that displays activation gating within this voltage range. There are 2 prominent candidate conductances that fulfill this criteria in CA1-PCs, namely HCN channels and Kv7 channels. The presence of HCN channels is reflected in the higher R_in_ seen at −74 mV than −80 mV (something known classically as anomalous rectification), as well as the voltage “sag” observed when negative current is injected into the cells to hyperpolarize them. We recently reported changes to voltage sag in a different model of tauopathy (Booth et al., 2016a, Booth et al., 2016b), although the lack of genotype-related differences in this parameter in this model argues against, but perhaps does not entirely rule out, changes to HCN channels in Tau35 mice.

Our observations are consistent with the presence of more active Kv7 channels in the Tau35 mice. These voltage-gated K^+^ channels, which are responsible for the so-called M-current, are tonically active at rest in CA1-PCs and are turned on by further depolarization. Our observations of a lower R_in_ in Tau35 at −74 mV could reflect either a larger total number of channels in Tau35 mice or alternatively a negative shift in the voltage dependence of channel opening. To date these have mainly been studied in the context of cancer epigenetics (Benoit et al., 2014). Notably, however, Tau35 mice have been shown to display increased GSK-3β activity (Bondulich et al., 2016), and this kinase is reported to modulate the gating properties of Kv7.2 channels via phosphorylation (Cooper et al., 2001, Singh et al., 1998, Wildburger and Laezza, 2012). In pyramidal neurons of the medial prefrontal cortex, inhibition of GSK-3β produces a hyperexcitability that mimics and occludes the effect of a Kv7 antagonist (Kapfhamer et al., 2010). Consequently, we hypothesize that in Tau35 mice activation of Kv7 channels by GSK-3β-dependent phosphorylation can modify excitability in CA1-PCs.

Our voltage clamp recordings comparing K^+^ currents in somatic macropatches from WT and Tau35 mice are certainly supportive of a negative shift in activation gating of a population of noninactivating voltage-gated K^+^ channels. However, we do not know what components of these currents, if any, result from activity of Kv7 channels. Some evidence indicates that Kv7 channels can be localized on the soma both with functional (Shah et al., 2011) and immunohistochemical measures (Cooper et al., 2001, Roche et al., 2002). However, these channels are thought to be more strongly localized on the axonal initial segment (AIS) and nodes of Ranvier.

**Fig. 9:**
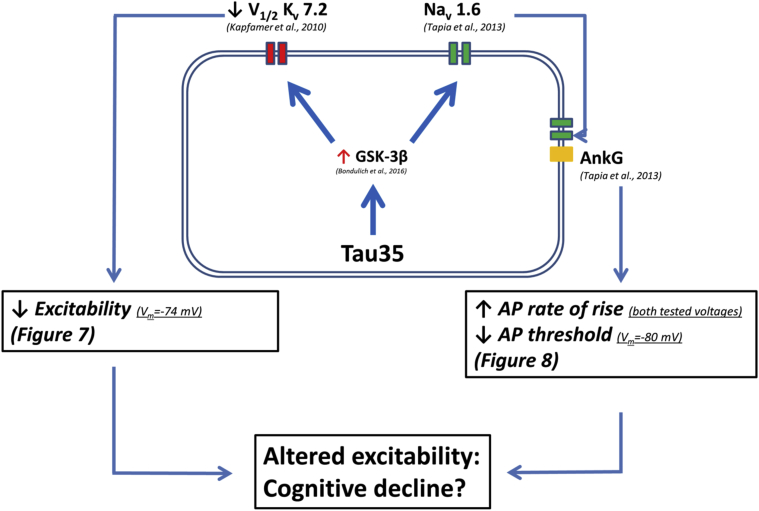
Schematic representation of the cascade of events potentially connecting Tau35 accumulation to altered intrinsic excitability of hippocampal CA1-PCs. Tau35 accumulation results in activation of GSK-3β (Bondulich et al., 2016), which has been reported to decrease the gating potential of K_v_7.2 channels and to increase Na_v_1.6 docking on the AIS via the AIS-associated protein AnkG. These events might be responsible for the reduced intrinsic excitability observed in Tau35 mice. Hyperpolarization of K_v_7.2 channels V_1/2_ could reduce input resistance and AP firing rate at depolarized potentials, whereas increased docking of Na_v_1.6 channels to the axonal initial segment could reduce the AP threshold and increase the AP maximal rate of rise. The causal relationship between these electrophysiological alterations and the cognitive decline observed in the Tau35 model of sporadic tauopathy remains to be clarified. Abbreviations: AIS, axonal initial segment; AnkG, ankyrin G; AP, action potential; GSK-3β, glycogen synthase kinase 3β; K_v_ 7.2, voltage-gated K^+^ channels 7.2; Na_v_ 1.6, voltage-gated Na^+^ channels 1.6; R_in_, input resistance; ROR, max rate of rise; Tau35, C-Terminal 35 kDa human tau fragment; Thr, Threshold; V_1/2_, half-activation potential; V_m_, membrane potential.

We also observed an increase in AP rate of rise in Tau35 mice. This was seen from both prestimulus membrane potentials examined. We also observed a hyperpolarization of the AP threshold in Tau35 animals but only when the cells were at −80 mV prior to the stimulus. However, the voltage dependence of AP threshold change between genotypes should be considered with caution, as one value among the −74 mV, Tau35 population appears to be an outlier and its removal would bring the AP threshold to be significantly hyperpolarized in Tau35 mice at both tested potentials. These alterations to AP properties could also result from the activity of GSK-3β, which has been shown to increase docking of Ankyrin-G, which results in elongation of the AIS and an increased surface density of Nav1.6 channels. In fact, GSK-3β has been shown to promote the elongation of the AIS and the reduction of the AIS distance from the soma via increased Ankyrin-G docking, changing the AP waveform properties and cell excitability (Tapia et al., 2013). Fig. 9 illustrates the mechanistic pathway potentially underlying our observations.

Previous work showed a striking increase of CA1-PCs I_h_-dependent resonance properties in rTg4510 mice (Booth et al., 2016b), particularly increased Peak Fr and Q, but no changes in impedance. Here, although no differences were observed in Peak Fr and Q, the overall impedance, measured as area under curve, was significantly smaller in Tau35, probably as a consequence of reduced input resistance at depolarized potentials, possibly mediated by I_M_ or other noninactivating K^+^ currents. This could be interesting evidence suggesting how different forms of pathogenic tau can differentially affect neuronal resonance.

The bidirectionality of excitability, depending on membrane potential, is an interesting observation. Although studies carried out on genetic models of frontotemporal dementia, such as rTg4510 mice, reveal an overall reduction of hippocampal network activity, presumably due to cell death (Booth et al., 2016b), other research works carried out on transgenic mice bearing a transgene associated to increased risk of sporadic frontotemporal dementia, reveal hippocampal hyperexcitability (Maeda et al., 2016, Siskova et al., 2014). Our observations reveal that single-cell excitability in CA1-PCs may change with membrane potential in Tau35 mice, presumably decreasing during the raising phase of the wave and vice-versa; however, to causally relate these results to network alterations, an in vivo study involving electrophysiological recordings preferably in behaving animals is required. In particular, it has been shown that the administration of the histone deacetylase (HDAC) inhibitor 4-phenyl butyrate in Tau35 mice results in the reduction of cognitive and motor impairment, GSK-3β activation and the associated hyperphosphorylation of tau protein; a key experiment, for exploring the mechanistic correlates of Tau35 altered electrophysiology described in this work, will be to test CA1-PC excitability and in vivo hippocampal network activity in 4-phenyl butyrate treated animals.

In summary, we have identified a range of neurophysiological consequences in hippocampal neurons associated with the Tau35 genotype. Although experimentally induced LTP is intact, we observed an increase and decrease of excitability of Tau35 CA1-PCs at polarized and depolarized membrane potentials, respectively. We hypothesize that this change is mediated by the phosphorylation of Kv7 and Nav1.6 channels, respectively, as increased GSK-3β activity is observed in Tau35 mice (Bondulich et al., 2016). A more detailed mechanistic characterization will be necessary to establish the causative role of GSK-3β in the electrophysiological alterations described in this work.

## Disclosure statement

The authors have no actual or potential conflicts of interest.
